# Temporal Characteristics of Radiologists' and Novices' Lesion Detection in Viewing Medical Images Presented Rapidly and Sequentially

**DOI:** 10.3389/fpsyg.2016.01553

**Published:** 2016-10-07

**Authors:** Ryoichi Nakashima, Yuya Komori, Eriko Maeda, Takeharu Yoshikawa, Kazuhiko Yokosawa

**Affiliations:** ^1^Department of Psychology, The University of TokyoTokyo, Japan; ^2^The University of Tokyo HospitalTokyo, Japan

**Keywords:** lesion detection, medical screening, stack viewing, rapid serial visual presentation, visual search in dynamic display, radiologist

## Abstract

Although viewing multiple stacks of medical images presented on a display is a relatively new but useful medical task, little is known about this task. Particularly, it is unclear how radiologists search for lesions in this type of image reading. When viewing cluttered and dynamic displays, continuous motion itself does not capture attention. Thus, it is effective for the target detection that observers' attention is captured by the onset signal of a suddenly appearing target among the continuously moving distractors (i.e., a passive viewing strategy). This can be applied to stack viewing tasks, because lesions often show up as transient signals in medical images which are sequentially presented simulating a dynamic and smoothly transforming image progression of organs. However, it is unclear whether observers can detect a target when the target appears at the beginning of a sequential presentation where the global apparent motion onset signal (i.e., signal of the initiation of the apparent motion by sequential presentation) occurs. We investigated the ability of radiologists to detect lesions during such tasks by comparing the performances of radiologists and novices. Results show that overall performance of radiologists is better than novices. Furthermore, the temporal locations of lesions in CT image sequences, i.e., *when* a lesion appears in an image sequence, does not affect the performance of radiologists, whereas it does affect the performance of novices. Results indicate that novices have greater difficulty in detecting a lesion appearing early than late in the image sequence. We suggest that radiologists have other mechanisms to detect lesions in medical images with little attention which novices do not have. This ability is critically important when viewing rapid sequential presentations of multiple CT images, such as stack viewing tasks.

## Introduction

### Examining specialized ability of experts

Experts exist in many domains, and they show high task performance levels in their expert domains (e.g., Chase and Simon, 1973; Voss et al., 1980; Schwaninger et al., 2005; Asano et al., 2008; Evans et al., 2011a). For example, master chess players or professional sports players are better at memorizing game-like arrays (i.e., meaningful materials) than novices (Chase and Simon, 1973; Voss et al., 1980). Additionally, a recent example has shown that medical experts (e.g., radiologists or cytologists) recognize (or memorize) medical images (e.g., mammograms or Pap smears) with greater accuracy than novices (Evans et al., 2011a). It is important to understand these experts' specialized cognitive abilities not only for cognitive science but also for understanding the applications of these abilities in individuals' routine tasks. This study focused on radiologists and examined their abilities in a medical image reading task.

Specifically, we compared abilities of radiologists with those of novices in a visual lesion search task. The typical medical image reading procedure consists of two basic processes: One process involves detection of a suspicious area, and the other process entails interpreting the gravity of a suspicious area, i.e., whether or not it represents a lesion (Krupinski, 2010). Although medical experts must exhibit superior skill throughout all stages of medical search, this study focuses on skills involved in the first stage, which involves a lesion detection process. This is because novices are not qualified to interpret the seriousness of the lesions.

The experimental task in this study focused on detection of a relatively obvious lesion. The suspicious areas include obvious locations as well as subtle or hidden locations such as lesions masked and/or overlapped by other anatomical features. Although many studies in radiology have discussed the detection of uncertain (i.e., not obvious) lesions (e.g., Evans et al., 2011b, 2013; see also Metz, 1978), visual search for obvious lesions is also an important issue (e.g., Carmody et al., 1981; Oestmann et al., 1988; Maeda et al., 2013; see also Kundel, 2006). Actually, missing the lesions in a search often occurs even when the lesions are relatively obvious (e.g., *scanning errors*: observers do not fixate on the lesions at all; see Kundel et al., 1978).

### Recent routine medical image readings

Recently, medical image reading includes not only the static image viewing such as mammography, ultrasound, and computed radiography, but also the dynamic image viewing such as sequential viewing of multiple slices of MRI (magnetic resonance imaging) and CT (computed tomography) images. Contemporary medical image reading tasks have increased enormously in size and complexity of routine screening due to rapid technological developments (Andriole et al., 2011). Today, viewing stacked CT images on a PC display is a routine practice in nearly all radiology departments (e.g., Copley et al., 2010; Drew et al., 2013). In this process, a radiologist typically scrolls quickly through a stack of 2-D CT images, which are thin slices of the 3-D volume of an organ. This is known as “stack viewing.” Although a number of studies have examined search strategies used in viewing single 2-D medical images (e.g., Carmody et al., 1981; Kundel et al., 1991; Ellis et al., 2006; Manning et al., 2006; Evans et al., 2013; Maeda et al., 2013; Nakashima et al., 2013, 2015), little is known about searches of 3-D medical images. Radiologists, who engage in stack viewing to detect lesions in an organ, usually scroll through the series of 2-D images very quickly at a more or less constant speed. However, upon finding a suspicious area in an image, their scrolling slows and sometimes reverses to view the image in more detail.

This brief overview of stack viewing highlights the fact that one axis of the 3-D volume image is a temporal axis in stack viewing. Accordingly, we can regard stack viewing as a type of visual search in a dynamic display. In stack viewing tasks, radiologists search for lesions appearing briefly accompanied by a transient signal (i.e., an abrupt appearance signal) that stand out among blood vessels and other organs appearing to move smoothly in an image sequence (apparent motion). Kunar and Watson (2011) suggested that some fundamental characteristics evident in visual searches of static images do not apply to visual searches in dynamic displays. As a result, visual search abilities of radiologists viewing sequentially presented medical images are not well understood (see Drew et al., 2013), although this is a problem that has critical implications for the welfare of society.

Previous studies have compared viewers' visual search strategies for detecting a target with a transient signal of the appearance in cluttered and dynamic displays. The results showed that a search strategy requiring passive viewing with few eye movements by an observer yields better target detection performance than a strategy requiring active scanning with many eye movements (e.g., Boot et al., 2006; Becic et al., 2007; see also Shapiro and Raymond, 1989). In a dynamic display, the continuous stimulus motion (i.e., motion itself) does not capture attention (cf. Abrams and Christ, 2003, 2005), but a transient signal by abrupt appearance of an object (i.e., object onset) does (cf. Yantis and Jonides, 1984). Therefore, a passive viewing strategy where observers' attention is captured by the target onset may be the most effective strategy in a dynamic visual search task.

In dynamic visual search tasks such as stack viewing, it may be easy to detect the target if the transient signal by abrupt appearance of a target captures attention. Although a continuous and smooth motion does not capture attention, the initiation of motion (i.e., motion onset) captures attention (e.g., Abrams and Christ, 2003, 2005). Considering the sequential image presentation, the moment of the initiation of the image presentation, the (apparent) motion onset signal by replacing a static image with another image should occur. In stack viewings of medical images, this motion onset occurs in the entire image simultaneously, because vessels exist everywhere in the medical image. We define this as a global apparent motion onset signal in this study. A motion onset signal has the potential for capturing attention even it if is task irrelevant (Kawahara et al., 2012). Thus, it is unclear whether observers can detect the target onset when the target appears at the beginning of a dynamic display where the global motion onset signal occurs. In other words, even if a strategy based on detection of the target onset signal is reliable, it can be jeopardized if the transient signal of a target appearance is occluded by the global motion onset signal that accompanies the beginning of each successive image slice in a dynamic display (e.g., an image sequence during stack viewing). That is, *when* a lesion appears in a sequence of CT images, either early or late in a sequence, is an important consideration for identifying factors influencing radiologists' efficiency in detecting lesions. For example, it becomes a critical issue in the medical field if radiologists show frequent misses of lesions that appear in early in an image sequence during stack viewing. This issue has not been addressed in previous studies, because the target occurred several seconds after the start of a trial (Boot et al., 2006; Becic et al., 2007).

### Purpose of this study

In summary, it is not clear whether medical experts perform better than novices in dynamic lesion search tasks, i.e., when medical images are presented sequentially as dynamic displays. This is pertinent, because dynamic lesion searches have become common in current medical image readings. Further, considering the dynamic lesion search task, it is also unclear whether the radiologists' lesion detection performances are systematically influenced by the temporal target location, i.e., the timing of a lesion's appearance in the sequence of medical images.

Accordingly, this study has three aims. First, we examined whether medical experts show reliably higher performance levels than novices in medical image reading tasks in dynamic display such as stack viewing tasks. That is, we aimed to replicate the results of previous studies using a medical image reading task in a static image (e.g., Evans et al., 2013; Nakashima et al., 2013, 2015), using another medical image reading task with a dynamic image presentation (cf. Kunar and Watson, 2011). Second, we examined whether radiologists can correctly detect the presence of a lesion even when the lesion appears early in the image sequence. As described above, the beginning of a sequential presentation of medical images generates a global apparent motion onset signal, which attracts visual attention. It is possible that a lesion appearing briefly at the beginning of such a sequence is difficult to detect. Third, related to the second aim, we confirm that novices would be more likely to miss a target lesion appearing early than late in a dynamic visual search display. If it is difficult to detect the transient signal of a lesion onset when the global apparent motion onset signal has also occurred, then lesion detection, at least for novices, should suffer especially when a target lesion appears early rather than later in sequence. In sum, we examined the visual search abilities of radiologists during the lesion detection in dynamic search task, by comparing performances of radiologists with those of novices.

In this experiment, the medical images were presented automatically. Strictly speaking, this is not identical to the routine stack viewing of medical screening tasks, because in the normal contexts radiologists can freely scroll through the stacks of images (Gur et al., 1994; Copley et al., 2010; Drew et al., 2013). However, this task has the advantage that we can rigorously control the presentation rate. It is noted that the presentation rate of 100 ms per image is not too fast compared to the radiologists' daily scroll speed (cf. Copley et al., 2010), and is relevant to the perception of apparent motion (see Movie S1).

## Materials and methods

### Ethics statement

This experiment was approved by the institutional review board of The University of Tokyo, and written informed consent was obtained from all participants. This experiment was conducted in accordance with the Declaration of Helsinki in the treatment of the participants.

### Participants

Twenty-seven novice young adults (age: 20–34 years) and seven radiologists from the University of Tokyo Hospital (age: 29–42 years; career experience: 3–18 years) completed this experiment. They were naive with respect to the purpose of the study. All participants reported that they had normal or corrected-to-normal vision (self-reports).

### Stimuli

**Figure 1:**
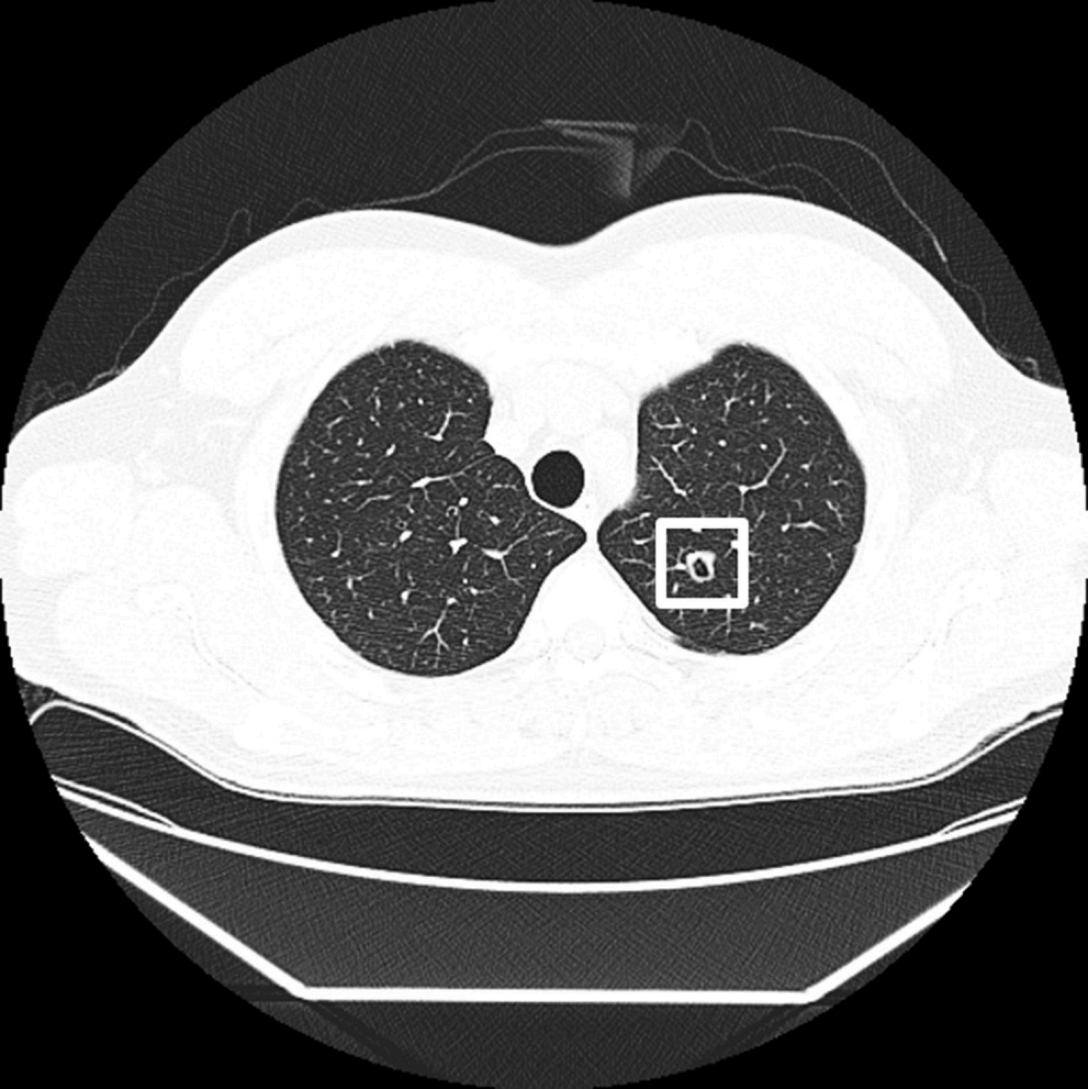
**An example of the background CT image and a target lesion image used in the experiments**. The target lesion is marked by a white frame, which was not presented in the experiment, in this figure.

We prepared 316 CT original sliced images of lungs (20.7 × 20.7 cm; 15° × 15°); they were image slices of healthy lungs of five de-identified people reviewed by radiologists in The University of Tokyo Hospital as containing no lesions (see Figure 1). Ten consecutive images were selected from the CT images to serve as a target-absent sequence. Each sequence included 10 CT images of the same person. We created 40 such target-absent sequences, allowing duplication of some image slices between the sequence stimuli. In addition to these stimulus sets, we created other 12 target absent sequences for “explanation trials.”

We prepared an image of a lesion “Cavitary Nodule” (see Erasmus et al., 2000; Honda et al., 2007), whose size was about 0.3° × 0.3° (Figure 1). The sizes of the lesions corresponded to about 5 × 5 mm when the size of one CT image was 20.7 × 20.7 cm. Eight images of Cavitary Nodule were served as targets. For this task, we used the lesion images pathologically diagnosed with lung cancer by radiologists in The University of Tokyo Hospital. We chose this lesion as a target in this study, based on the fact that radiologists can detect a target lesion well especially when they search for serious lesions (Nakashima et al., 2015). The lesion image looked like an irregularly bordered white nodule containing a small black hole inside, which was distinguishable from images of background blood vessels that appeared as white filled ovals or dots. Medically, this image strongly suggests lung cancer, although differential diagnosis includes several infectious and non-infections process. To create a lesion-present image, we first randomly chose one of the images from the 2nd to the 9th image in each target-absent sequence. The probability that the target presented in the each image (i.e., the 2nd–9th image) was equal. A target lesion image was placed at a random but anatomically feasible spatial location (i.e., to avoid the lesion overlapping with blood vessels, bronchi and other organs) within the selected image, using Photoshop (Adobe). A target-present sequence comprised nine of the original target-absent displays with one of these replaced by its corresponding lesion-present CT image. As a result, we made 40 target-present sequences. The lesion appeared in the first (i.e., 2nd–3rd image), second (i.e., 4th–5th image), third (i.e., 6th–7th image), and fourth (i.e., 8th–9th image) quarters of sequences equally often in the target-present sequences. Two stimulus sets were made in which 20 target-absent sequences and 20 target-present sequences. Strictly speaking, the image sequences are not the same as the realistic medical sequence, because lesions have depth and appear in multiple successive slices in a sequence, rather than only in a single slice. However, the present method has the advantage that we can rigorously control the presentation duration of a lesion. We created additional 6 target present sequences using the half of the 12 sequences for explanation trials. Radiologists in The University of Tokyo Hospital, who did not participate in the experiment, supervised stimulus construction, and confirmed that the images looked realistic to the radiologists.

### Apparatus

Presentation of stimuli and recording of responses were controlled by Matlab with the Psychophysics Toolbox extensions (Brainard, 1997; Pelli, 1997). Stimuli were displayed at a resolution of 1024 × 768 pixels on a 22-inch monitor (refresh rate: 60 Hz).

### Procedure

Participants were seated in front of a computer monitor in a dark room (viewing distance was 77 cm fixed by a forehead and chin rest). Before the experiment, participants received an explanation of target lesions (i.e., “a small open oval”) and were shown examples of background and target lesion images. It is noted that radiologists recognized the name of the target lesion “Cavitary Nodule” before the experimental session, because they were familiar with the lesion. Thus, we told the name of the target lesion to the radiologists. We did not tell novices the name of the lesion, because the three authors, who did not major in radiology, did not recognize the target lesion even if they were told the name before this study. Then, the participants received 12 explanation trials to familiarize with the experimental paradigm, target appearance and presentation timing (500 ms/image). We did not include these data in the analysis.

**Figure 2:**
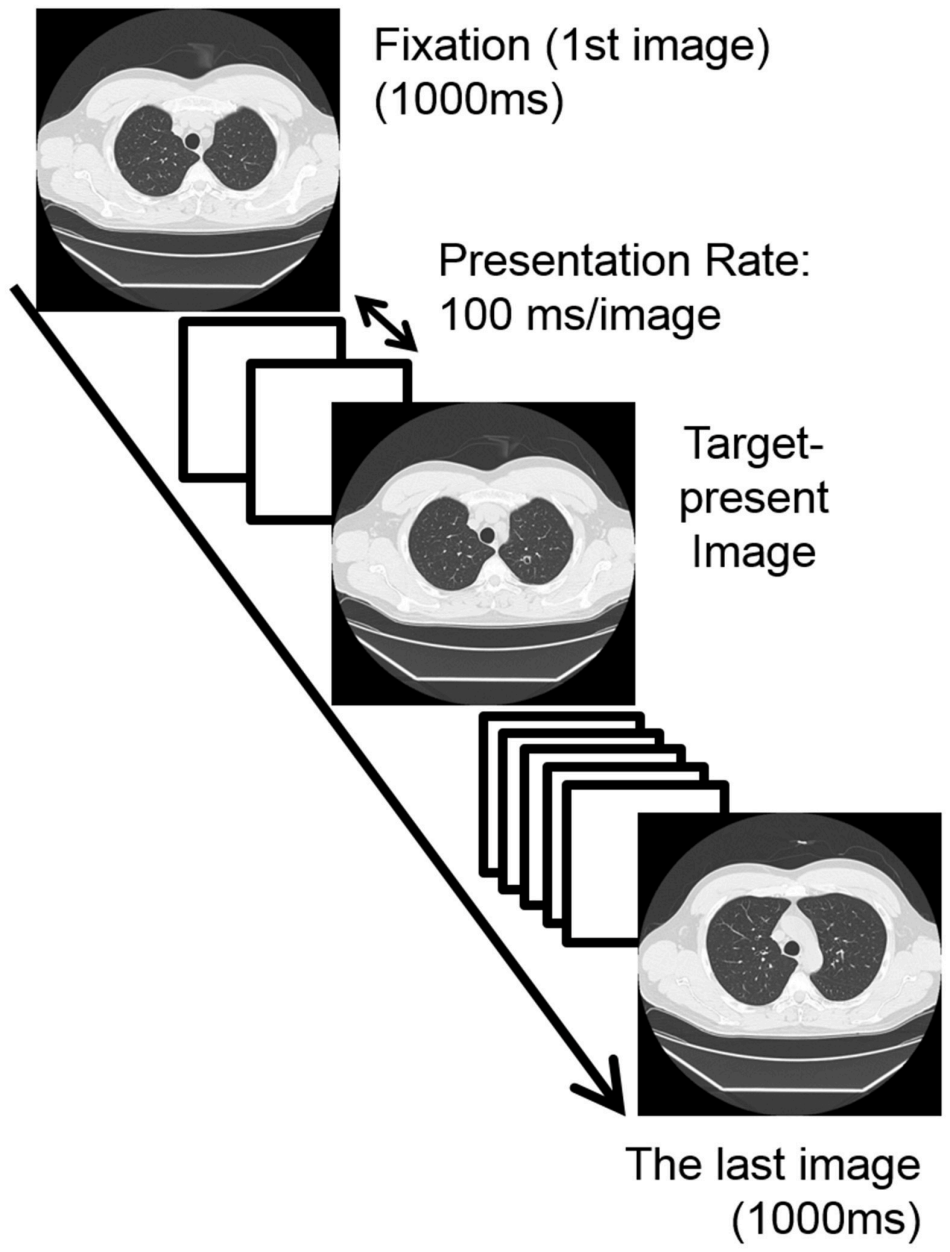
**An example of the sequence of one trial in the experimental session where the presentation rate (i.e., stimulus onset asynchrony; SOA) was 100 ms without the inter-stimulus interval**. White squares in this figure indicate the target absent images. In this case, the target lesion was presented on the 4th image of the sequence (2nd bin). In the “practice sessions,” the presentation rates were 500 ms/image (practice-session-“slow”) and 167 ms/image (practice-session-“fast”).

Participants received two practice sessions and then one experimental session. The tasks were the same in all sessions except for the image presentation duration. One image sequence was presented in one trial. In experimental session, on each trial, the first image of one sequence was presented as a fixation image (1000 ms); next, eight images were presented with 100 ms duration each with no inter-stimulus interval; then the last image was presented for 1000 ms (Figure 2). The sequence appeared to be as a dynamic movie created through the apparent motion signal based on the fact that the locations of vessels were different in each CT image slice (see Movie S1). Participants were instructed to judge, as accurately as possible, whether the target was present or absent within an image sequence (in one of the 2nd–9th images) by pressing one of the response keys (2AFC task). They were allowed to respond even during the presentation of the image sequence if they found the target. They received 40 trials (20 target-present and 20 target-absent trials) where no feedback was given for their responses. The inter-trial interval was 1 s. One of the stimulus sets was assigned to the experimental session. The other set was assigned to the “practice session” twice. Two “practice sessions” including 40 trials were conducted, manipulating the presentation rate: first 500 ms/image for familiarizing them with the sequential presentation, named the practice-session-“slow,” and then 167 ms/image for familiarizing them with the relatively fast, but slower than that in the experimental session, sequential presentation, named the practice-session-“fast.” It is noted that we analyzed the data of practice-session-“slow.”

## Results and discussion

### Data analysis

In this study, we used d-prime in the signal detection theory (i.e., detection sensitivity), based on the detection rate (i.e., rate of correct “target present” responses) and the false alarm rate (i.e., rate of false “target present” responses in target absent trials), as the dependent variable. A typical visual search experiment in experimental psychology presents static visual stimuli that remain visible until observers respond to them; the dependent variable is time-to-detect a target (e.g., Treisman and Gelade, 1980; Treisman, 1988; Nakashima and Yokosawa, 2013; see also Wolfe, 1998). By contrast, in other tasks, visual stimuli are presented with brief exposure durations, and the dependent variable is detection accuracy or detection sensitivity (e.g., Inui et al., 1978; Bergen and Julesz, 1983; Sagi and Julesz, 1985; Braun and Sagi, 1990). It is appropriate to measure the detection sensitivity in the present task where the images are presented sequentially, because each image is presented briefly.

To compare the overall performances between the groups, we conducted the Welch's *t*-test because of unequal participant groups. The Welch's *t*-test does not assume that the standard deviations of the data groups are equal, and therefore it is suited to the analysis of the present data (i.e., unequal sample size). Then, an additional one-way Analysis of Variance (ANOVA), where the data of the groups were analyzed separately, was conducted to examine the effect of the temporal target location in a CT image sequence (i.e., when a lesion appears in a sequence of sliced images of lung) on the lesion detection performance in each group.

### Group comparison

The mean d-primes were 2.81 in radiologists and 1.86 in novices. Welch's *t*-test revealed the d-prime was higher in radiologists than in novices, *t*_(11)_ = 5.23, *p* < 0.001. This result suggests that the medical experts show the higher performance than novices in their expert domain, and is consistent with the previous studies (e.g., Evans et al., 2013; Nakashima et al., 2013, 2015).

### Detection performance based on the temporal target location in radiologists and novices

**Figure 3:**
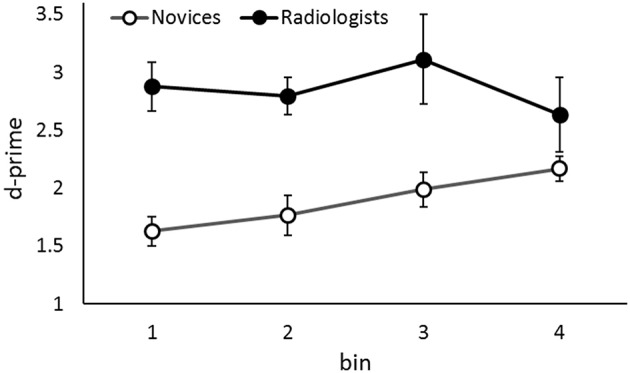
**The d-primes in the fast presentation rate condition as a function of the group and the temporal location of the target in the sequence**. Error bars indicate 95% confidence intervals. Although the data of two groups are shown simultaneously, the data were analyzed separately.

We examined whether or not temporal locations of lesions in a CT image sequence influence lesion detection rates of novices and radiologists. To assess this, we divided the detection performance data (i.e., d-prime) into four bins based on the temporal lesion location condition (five trials per bin); these are labeled 1st, 2nd, 3rd, and 4th bins (Figure 3). A one-way ANOVA was conducted on d-prime with Temporal Target Location as a within-participants factor.

Results revealed a significant main effect of Temporal Target Location in novices, *F*_(3, 78)_ = 6.03, *p* = 0.001, η_*p*_^2^ = 0.19. The tendency to perform better in lesion detection in the latter of the sequence was evident. That is, performance was better in the 4th bin than in the 1st and 2nd bins, *p*s < 0.03, and better in the 3rd bin than in the 1st bin, *p* = 0.03. This leads to the interpretation that novices fail to detect a target lesion appeared early in the sequence when the global apparent motion onset signal occludes the transient signal of the target appearance.

In contrast, although the performance appeared to be worse in the 4th bin than the other bins (see Figure 3), there were no statistical differences among the performances in the bins in radiologists, *F*_(3, 18)_ = 1.46, *p* = 0.26, η_*p*_^2^ = 0.19. Conservatively, we did not suggest that radiologists' lesion detection performance is influenced by the temporal lesion location, i.e., when a lesion appears in a sequence, in contrast to the novices.

One might wonder that it is possible that the performances of radiologists did not differ among the bins but those of novices did, just because the sample size of novices were larger than radiologists. To confirm that the results of radiologists did not derive from low statistical power, we additionally conducted a two-way mixed factorial ANOVA for the data of first 7 novices who participated in this experiment and 7 radiologists. The mean d-primes of the 7 novices were 1.46, 1.93, 1.99, and 2.31 in 1st, 2nd, 3rd, and 4th bins. An ANOVA revealed a significant main effect of Group, *F*_(1, 12)_ = 11.79, *p* = 0.005 η_*p*_^2^ = 0.49, but not of Temporal Target Location, *F*_(3, 36)_ = 1.77, *p* = 0.17, η_*p*_^2^ = 0.13. Furthermore, the interaction between these two factors was significant, *F*_(3, 36)_ = 3.46, *p* = 0.03, η_*p*_^2^ = 0.22. In novices, the tendency to perform better in lesion detection in the latter portion of a sequence was evident. That is, performance was better in the 4th bin than in the 1st bin, *p* = 0.01. In contrast, there were no statistical differences among performance levels in bins for radiologists, *p* = 0.26. These results are very similar to the results described above, i.e., the data of 27 novices and 7 radiologists. Even when the novices are few, the temporal target location did influence their target detection performance. Therefore, the matter of statistical power is not likely to fully explain the results that temporal target location influenced only the performance of novices in this study. In addition, the significant interaction indicates that radiologists can detect the presence of a lesion even in the situation where the lesion appears briefly, and that their lesion detection performance is more stable against the temporal target location in a sequence than novices.

### Lesion detection performance in slow presentation task

**Figure 4:**
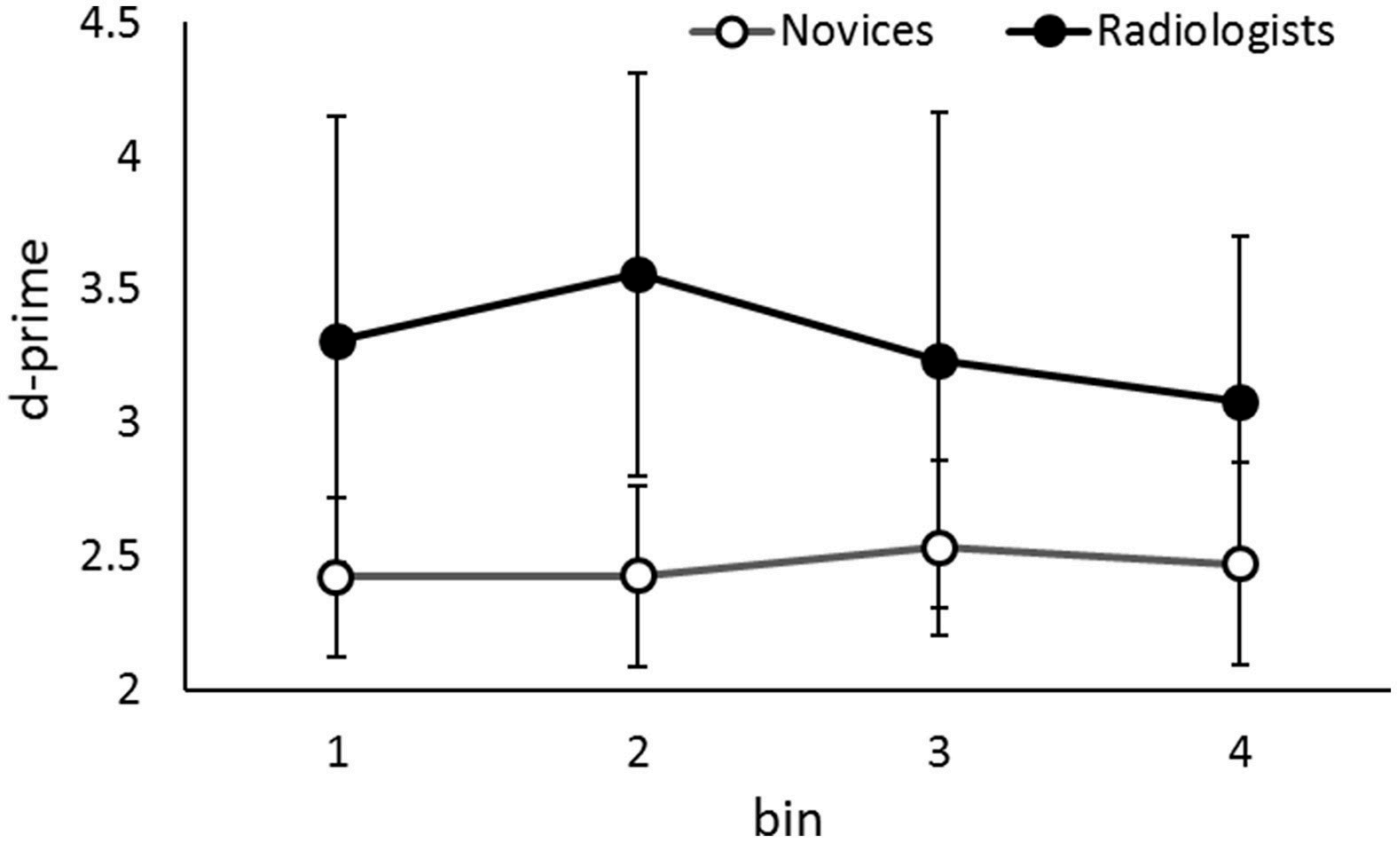
**The d-primes in the slow presentation rate condition (i.e., the practice-session-“slow”) as a function of the group and the temporal location of the target in the sequence**. Error bars indicate 95% confidence intervals. Although the data of two groups are shown simultaneously, the data were analyzed separately.

We analyzed the performance of both group when the presentation rate was slow, although it was not our main purpose in this study, to check whether both novices and radiologists can detect lesion accurately when they have long time to view each image. We examined the effect of the temporal target location in the slow sequential image presentation where observers could recognize the detailed information (e.g., Tversky and Sherman, 1975; Loftus et al., 1983). We analyzed the data in the practice-session-“slow” (presentation rate: 500 ms/image, 40 trials), where the stimuli were different from those used in the experimental session. Figure 4 shows the results. Again, the overall performance (d-prime) was higher in radiologists (3.19) than in novices (2.38), *t*_(22)_ = 5.80, *p* < 0.001. This is consistent with the results of previous studies (e.g., Evans et al., 2013; Nakashima et al., 2013, 2015). An additional ANOVA revealed that the main effects of Temporal Target Location were not significant in either group, novice: *F*_(3, 78)_ = 0.19, *p* = 0.89, η_*p*_^2^ = 0.008, radiologist: *F*_(3, 18)_ = 0.75, *p* = 0.53, η_*p*_^2^ = 0.11. In contrast to the results in fast presentation rate task, the performances did not differ based on the temporal target location, i.e., when a lesion appeared in an image presentation sequence. Thus, novices can detect lesions relatively accurately whenever the lesion appeared in a sequence, if they have enough time to view each image.

## General discussion

### Summary of results

Although viewing methods where observers view many sliced images through the body sequentially have become widespread in recent medical image reading, little is known about how lesion detection is accomplished in this type of task. We examined the performance of lesion detection by radiologists and novices when they viewed the rapid sequential presentation of CT images, simulating the period before radiologists find a suspicious area in CT images. In terms of the three aims of the current study, we obtained the following results.

First, overall lesion detection was better in radiologists than in novices. This confirms the suggestions in previous studies that medical experts (e.g., radiologists) show high performance in their specific domain (e.g., Evans et al., 2013; Nakashima et al., 2013, 2015), even when image presentations are dynamic. Second, the temporal locations of lesions in a rapid CT image sequence had little influence on the lesion detection performance in radiologists. Third, in contrast to radiologists, the temporal location of lesions had a significant effect on the performance of novices, showing the lower target detection performance when the lesion appeared early in the sequence. We can interpret this difficulty for novices in detecting early targets in the sequence as being due to the occlusion of the transient signal of the target by the global apparent motion onset signal. Based on the second and third results, we indicated the radiologists' specialized ability during a dynamic visual search display, namely the temporal stability of lesion detection.

### Lesion detection in a dynamic display and radiologists' specialized ability

Results show that radiologists can detect lesions accurately whenever a lesion appears in a CT image sequence, whether the sequence is presented at rapid or a slow rate. In a rapid sequence presentation, even though the transient signal of the target appearance is occluded by the global apparent motion onset signal capturing attention (Abrams and Christ, 2003, 2005), radiologists detect the presence of a lesion with high accuracy. In this case, the attentional mechanism to find a lesion, e.g., the involuntary attentional capture, does not function well. Further, in this study, the target-present CT image was created by adding a lesion image to a normal CT image of healthy lung. Thus, participants, even well-trained professional radiologists, could not predict whether a lesion would appear in the sequence at all when they viewed the 1st CT (i.e., fixation) image. Much more, they could not predict where a lesion would appear in a CT image. Thus, it is useless (or impossible) to move their eyes to a probable area of the lesion appearance beforehand. In sum, the attentional mechanism or the beforehand gaze position strategy cannot provide a sufficient explanation of these results.

Instead of the attentional mechanism to focus attention on a target lesion, which usually functions in conventional visual search processing, other mechanisms appear to function in this situation. We propose two possible mechanisms in this study. First, radiologists have a precise target lesion representation (i.e., a good target template) for efficient lesion search. Previous visual search literatures have suggested that visual representation for a target facilitates visual search in static displays (e.g., Luria and Strauss, 1975; Cristie and Klein, 1995; Vickery et al., 2005; Bravo and Farid, 2009; Malcolm and Henderson, 2009; Hout and Goldinger, 2015). In addition, our recent study implies that radiologists show high detection sensitivities especially during searching for serious lesions (Nakashima et al., 2015). It is possible that radiologists can detect lesions during a difficult lesion search situation based on their acquisition of precise target templates for certain lesions. Assuming such templates have a strong influence on the target perception enough to be perceived preferentially or perhaps with little attention (cf. to be “pop-out” in the search display), radiologists can detect lesions during a difficult lesion search situation based on their learned precise target templates. By contrast, novices do not operate with such refined templates.

Second, radiologists detect lesions not only by focusing attention on a target lesion but also by extracting global information (e.g., whole structures and statistical regularities) indicating whether a CT image includes lesions or not (Evans et al., 2013). Previous studies have shown that medical experts (radiologists and cytologists) show above chance lesion detection performance in mammograms and cytology slides even though the image is presented very briefly (Kundel and Nodine, 1975; Carmody et al., 1981; Evans et al., 2013). People can pick up the general meaning or “gist” of a natural scene when they view the image briefly (e.g., Potter and Faulconer, 1975; Intraub, 1981) by extracting global information with little attention (cf. Li et al., 2002, 2005; Rousselet et al., 2002; but see Cohen et al., 2011). Radiologists should be able to judge whether a lesion exists or not (i.e., normal or abnormal) even when the lesion was presented early in the CT image sequence, if an attention-free lesion detection mechanism could compensate for interference with the attentional mechanism (i.e., to catch the transient signal of target onset). Because medical experts have acquired a global impression of the normality/abnormality of a medical image though training, which Evans et al. (2013) named “a trained specialization of gist processing,” novices of course cannot use this processing.

We cannot conclude which mechanism is better to explain the results, because our experiment was not conducted to examine these mechanisms. This issue should be examined in detail in further research. However, it should be noted that radiologists see lesion images in the context of medical readings; that is, they usually see lesions within a medical image, rather than abstracted from such a context. Therefore, we indicate that both target representations and global information of medical images should be important for the radiologists' lesion detection.

Although there are differences between this experimental task and real-world medical search, this study was designed to control for possible extraneous variables. First, the presentation rate of the image sequence was the constant in this experiment in order to control the image viewing time. This differs from the daily medical routine where radiologists can scroll through the stacks of images freely with a preferred speed. There can be differences in the medical viewing performance between the two tasks (Gur et al., 1994), and therefore, it is necessary to consider this issue in order to clarify the ideal image presentation rate for each radiologist. Second, a lesion appeared only in a single slice in this experiment in order to control the target viewing time. This differs from the daily medical routine where a lesion appears in multiple slices because of their depth. Thus, it is possible to lesion detection is more difficult in this experiment than in a daily task. Third, we focused on the lesion detection when the lesion appears early in a sequence consisting of only 10 images (per trial). In contrast, in daily stack viewing task radiologists view much more images than used in this experimental task. Previous studies have shown that people can hardly maintain a cognitive intent to orient attention toward a target intermittently over a prolonged period of time even if they should do this (vigilance decrement; cf. Davies and Parasuraman, 1982; Warm et al., 2008). Actually, the present results show that detection performance becomes lower when the lesion appears late in the sequence, although it is not significant. It is necessary to examine in detail the lesion detection performance not only when the lesion appears early but also very late in the sequence.

### Lesion search of novices in sequential image presentation task

The present results show that novices' lesion detection is poor when the lesions appear early in the sequence but that it improves when they appear late in the sequence. This can be interpreted as the suggestion that novices fail to detect a target lesion near the beginning of sequence when a global apparent motion signal occludes the transient signal of the target appearance. That is, the attentional mechanism for detecting a target lesion does not function well early in the sequence, whereas it does function adequately late in a sequence. This is a typical result of “attentional awakening” effect in a rapid serial visual presentation (RSVP) task (Ariga and Yokosawa, 2008; Kranczioch and Bryant, 2011). In attentional awakening experiments, only one target appears in an RSVP sequence, rather than two targets in classical attentional blink experiments (see Shapiro et al., 1997), and the identification of the target is impaired when it occurs early in this sequence. The previous studies indicated that this effect shows that observers can only gradually modulate their temporal attentional orienting to the stimuli presented sequentially. That is, the preparation of the temporal attention to synchronize with the stimuli presentation takes time, and the visual processing (e.g., target identification) become inefficient or incomplete during the attentional preparation. Based on the results of our experimental task which is similar to the attentional awakening experiment, we suggest that one of the reasons for the occurrence of attentional awakening, in addition to the suggestion in previous studies, can be that the target signal to be detected is masked by the signal of the sequence onset.

The novices' expectation of a target appearance may explain the results that novices detect lesions better when the lesions appear late in the sequence (i.e., expectation hypothesis). If observers do not see a lesion in the early part of the sequence, they may build up expectancy for a target appearance over the course of the trials. With high expectation of target appearances, they can detect a lesion when it appears late in the sequence. We cannot reject this explanation completely by the present results. Further research is necessary to address this issue. However, it should be noted that this explanation does not conflict with our suggestion that the global apparent motion signal occludes the transient signal of the target appearance, and thereby interferes with the target detection. That is, the premise of the expectation hypothesis holds that novices do not see a target early in the sequence.

Further, the detection performance in the slow presentation rate condition (i.e., the practice-session-“slow”) was somewhat high and robust over the temporal lesion location in a presentation sequence. This indicates that novices can detect lesions to some extent when they have sufficient time to view a CT image with focused attention even if the global apparent motion onset signal occludes the signal of a target appearance. Thus, novices' failure in detecting lesions can become obvious when an image is presented briefly.

### Limitation of the present study for application

As described above, because of certain differences between the present experimental task and ordinary daily task, and the small size of participants and trials, it may be difficult to generalize these results to the daily medical routine task. However, our results showed that the lesion detection performance of radiologists in a dynamic display (e.g., stack viewing) is more stable against the temporal lesion location in a sequence than that of novices. We believe that our findings provide clues to clarify the characteristics of radiologists' strategies for lesion detection. To examine our suggestion further, more detailed experiments, with larger sample sizes, are necessary in the future research.

## Conclusion

Medical experts (e.g., radiologists in this study) show remarkably high performance levels in a task that is common in their domain, i.e., a medical image reading task where images are presented sequentially. Radiologists can detect lesions well, even in difficult conditions where novices detect such lesions poorly.

Radiologists can correctly detect the presence of a lesion, even when it occurs early in a sequence of images. That is, radiologists can detect lesions whenever the lesions are presented in a CT image sequence, even when the sequence of images is presented rapidly. Therefore, radiologists must rely on the mechanisms requiring minimal attention for lesion detection in a medical image, in addition to their normal capacity to rely on focal attention for target lesion. Possible mechanisms we proposed are the lesion detection by using a precise target lesion representation and by extracting global information. These mechanisms may be particularly useful when radiologists view a large number of CT images sequentially as in a routine screening, because they enable the rapid and efficient lesion detection.

Novices do miss lesions appearing early in a CT image sequence, when the transient signal of the lesion appearance is occluded by a global apparent motion onset signal. In contrast, if the image sequence is presented slowly, novices can detect lesions whenever a lesion appears in the sequence, because they have enough time to view each image. That is, novices detect lesions in a CT image mainly through attentive processing in the search strategy (e.g., focusing attention on a target location or attentional capture by a signal of the target appearance) suitable for a dynamic display.

In summary, radiologists have a specialized ability for detecting lesions in medical images which novices lack, namely the temporal stability of lesion detection. This ability is responsible for the difference in the lesion detection performance between radiologists and novices, and is very useful when they viewed many medical images in a short period of time.

## Author contributions

RN, YK, EM, TY, and KY designed this work. RN and YK performed the experiment and analyses. RN, YK, EM, TY, and KY wrote the manuscript.

## Funding

This study was supported by JSPS KAKENHI Grant Number JP16K17368 to RN.

### Conflict of interest statement

The authors declare that the research was conducted in the absence of any commercial or financial relationships that could be construed as a potential conflict of interest.

## Supplementary material

Movie S1 **Examples of the sequential presentation**. First, SOA 500 ms condition (i.e., practice-session-“slow” session) and then SOA 100 ms condition (i.e., experimental session).

## References

[B1] Abrams R. A. Christ S. E. (2003). Motion onset captures attention. Psychol. Sci. 14, 427–432. 10.1111/1467-9280.01458 12930472

[B2] Abrams R. A. Christ S. E. (2005). The onset of receding motion captures attention: comment on Franconeri and Simons (2003). Percept. Psychophys. 67, 219–223. 10.3758/BF03206486 15971686

[B3] Andriole K. P. Wolfe J. M. Khorasani R. Treves S. T. Getty D. J. Jacobson F. L.. (2011). Optimizing analysis, visualization, and navigation of large image data sets: one 5000-section CT scan can ruin your whole day. Radiology 259, 346–362. 10.1148/radiol.11091276 21502391 PMC6939959

[B4] Ariga A. Yokosawa K. (2008). Attentional awakening: gradual modulation of temporal attention in rapid serial visual presentation. Psychol. Res. 72, 192–202. 10.1007/s00426-006-0100-4 17106706

[B5] Asano M. Kanaya S. Yokosawa K. (2008). Proofreaders show a generalized ability to allocate spatial attention to detect changes. Psychologia 51, 126–141. 10.2117/psysoc.2008.126

[B6] Becic E. Kramer A. F. Boot W. R. (2007). Age-related differences in visual search in dynamic displays. Psychol. Aging 22, 67–74. 10.1037/0882-7974.22.1.67 17385984

[B7] Bergen J. R. Julesz B. (1983). Rapid discrimination of visual patterns. IEEE Trans. Syst. Man. Cybern. 13, 857–863. 10.1109/TSMC.1983.6313080

[B8] Boot W. R. Kramer A. F. Becic E. Wiegmann D. A. Kubose T. (2006). Detecting transient changes in dynamic displays: the more you look, the less you see. Hum. Factors 48, 759–773. 10.1518/001872006779166424 17240723

[B9] Brainard D. H. (1997). The psychophysics toolbox. Spat. Vis. 10, 443–446. 10.1163/156856897X00357 9176952

[B10] Braun J. Sagi D. (1990). Vision outside the focus of attention. Percept. Psychophys. 48, 45–58. 10.3758/BF03205010 2377439

[B11] Bravo M. J. Farid H. (2009). The specificity of the search template. J. Vis. 9, 34.1–34.9. 10.1167/9.1.34 19271904

[B12] Carmody D. P. Nodine C. F. Kundel H. L. (1981). Finding lung nodules with and without comparative visual scanning. Percept. Psychophys. 29, 594–598. 10.3758/BF03207377 7279589

[B13] Chase W. G. Simon H. A. (1973). Perception in chess. Cogn. Psychol. 4, 55–81. 10.1016/0010-0285(73)90004-2

[B14] Cohen M. A. Alvarez G. A. Nakayama K. (2011). Natural-scene perception requires attention. Psychol. Sci. 22, 1165–1172. 10.1177/0956797611419168 21841149

[B15] Copley S. J. Bryant T. H. Chambers A. A. Harvey C. J. Hodson J. M. Graham A.. (2010). Observer accuracy in the detection of pulmonary nodules on CT: effect of cine frame rate. Clin. Radiol. 65, 133–136. 10.1016/j.crad.2009.05.016 20103435

[B16] Cristie J. Klein R. (1995). Familiarity and attention: does what we know affect what we notice? Mem. Cogn. 23, 547–550. 10.3758/BF03197256 7476240

[B17] Davies D. R. Parasuraman R. (1982). The Psychology of Vigilance. London: Academic Press.

[B18] Drew T. Vo M. L. Olwal A. Jacobson F. Seltzer S. E. Wolfe J. M. (2013). Scanners and drillers: characterizing expert visual search through volumetric images. J. Vis. 13, 3.1–3.13. 10.1167/13.10.3 23922445 PMC3736761

[B19] Ellis S. M. Hu X. Dempere-Marco L. Yang G. Z. Wells A. U. Hansell D. M. (2006). Thin-section CT of the lungs: eye-tracking analysis of the visual approach to reading tiled and stacked display formats. Eur. J. Radiol. 59, 257–264. 10.1016/j.ejrad.2006.05.006 16829011

[B20] Erasmus J. J. Connolly J. E. McAdams H. P. Roggli V. L. (2000). Solitary pulmonary nodules: Part I. Morphologic evaluation for differentiation of benign and malignant lesions. Radiographics 20, 43–58. 10.1148/radiographics.20.1.g00ja0343 10682770

[B21] Evans K. K. Cohen M. A. Tambouret R. Horowitz T. Kreindel E. Wolfe J. M. (2011a). Does visual expertise improve visual recognition memory? Atten. Percept. Psychophys. 73, 30–35. 10.3758/s13414-010-0022-5 21258906 PMC3140200

[B22] Evans K. K. Evered A. Tambouret R. H. Wilbur D. C. Wolfe J. M. (2011b). Prevalence of abnormalities influences cytologists' error rates in screening for cervical cancer. Arch. Pathol. Lab. Med. 135, 1557–1560. 10.5858/arpa.2010-0739-OA 22129183 PMC3966132

[B23] Evans K. K. Georgian-Smith D. Tambouret R. Birdwell R. L. Wolfe J. M. (2013). The gist of the abnormal: above-chance medical decision making in the blink of an eye. Psychon. Bull. Rev. 20, 1170–1175. 10.3758/s13423-013-0459-3 23771399 PMC3851597

[B24] Gur D. Good W. F. Oliver J. H. Thaete T. L. Baron R. L. Federle M. P.. (1994). Sequential viewing of abdominal CT images at varying rates. Radiol 191, 119–122. 10.1148/radiology.191.1.8134556 8134556

[B25] Honda O. Tsubamoto M. Inoue A. Johkoh T. Tomiyama N. Hamada S.. (2007). Pulmonary cavitary nodules on computed tomography: differentiation of malignancy and benignancy. J. Comput. Assist. Tomogr. 31, 943–949. 10.1097/RCT.0b013e3180415e20 18043361

[B26] Hout M. C. Goldinger S. D. (2015). Target templates: the precision of mental representations affects attentional guidance and decision-making in visual search. Atten. Percept. Psychophys. 77, 128–149. 10.3758/s13414-014-0764-6 25214306 PMC4286498

[B27] Intraub H. (1981). Rapid conceptual identification of sequentially presented pictures. J. Exp. Psychol. Hum. 7, 604–610. 10.1037/0096-1523.7.3.604

[B28] Inui T. Kawato M. Suzuki R. (1978). The mechanism of mental scanning in foveal vision. Biol. Cybern. 30, 147–155. 10.1007/BF00337143 708797

[B29] Kawahara J. Yanase K. Kitazaki M. (2012). Attentional capture by the onset and offset of motion signals outside the spatial focus of attention. J. Vis. 12, 10.1–10.13. 10.1167/12.12.10 23161003

[B30] Kranczioch C. Bryant D. (2011). Attentional awakening, resource allocation and the focus of temporal attention. Neuroreport 22, 161–165. 10.1097/WNR.0b013e3283438b76 21304326

[B31] Krupinski E. A. (2010). Current perspectives in medical image perception. Atten. Percept. Psychophys. 72, 1205–1217. 10.3758/APP.72.5.1205 20601701 PMC3881280

[B32] Kunar M. A. Watson D. G. (2011). Visual search in a multi-element asynchronous dynamic (MAD) world. J. Exp. Psychol. Hum. 37, 1017–1031. 10.1037/a0023093 21500946

[B33] Kundel H. L. (2006). History of research in medical image perception. J. Am. Coll. Radiol. 3, 402–408. 10.1016/j.jacr.2006.02.023 17412094

[B34] Kundel H. L. Nodine C. F. (1975). Interpreting chest radiographs without visual search. Radiology 116, 527–532. 10.1148/116.3.527 125436

[B35] Kundel H. L. Nodine C. F. Carmody D. P. (1978). Visual scanning, pattern recognition, and decision-making in pulmonary nodule detection. Invest. Radiol. 13, 175–181. 10.1097/00004424-197805000-00001 711391

[B36] Kundel H. L. Nodine C. F. Toto L. (1991). Searching for lung nodules – The guidance of visual scanning. Invest. Radiol. 26, 777–781. 10.1097/00004424-199109000-00001 1938287

[B37] Li F.-F. VanRullen R. Koch C. Perona P. (2002). Natural scene categorization in the near absence of attention. Proc. Natl. Acad. Sci. U.S.A. 99, 9596–9601. 10.1073/pnas.092277599 12077298 PMC123186

[B38] Li F.-F. VanRullen R. Koch C. Perona P. (2005). Why does natural scene categorization require little attention? Exploring attentional requirements for natural and synthetic stimuli. Vis. Cogn. 12, 893–924. 10.1080/13506280444000571

[B39] Loftus G. R. Nelson W. W. Kallman H. J. (1983). Differential acquisition rates for different types of information from pictures. Q. J. Exp. Psychol. 35A, 187–198. 10.1080/14640748308402124 6681183

[B40] Luria S. M. Strauss M. S. (1975). Eye movements during search for coded and uncoded targets. Percept. Psychophys. 17, 303–308. 10.3758/BF03203215

[B41] Maeda E. Yoshikawa T. Nakashima R. Kobayashi K. Yokosawa K. Hayashi N.. (2013). Experimental system for measurement of radiologists' performance by visual search task. SpringerPlus 2:607. 10.1186/2193-1801-2-607 24294550 PMC3838537

[B42] Malcolm G. L. Henderson J. M. (2009). The effects of target template specificity on visual search in real-world scenes: evidence from eye movements. J. Vis. 9, 8.1–8.13. 10.1167/9.11.8 20053071

[B43] Manning D. Barker-Mill S. Donovan T. Crawford T. (2006). Time-dependent observer errors in pulmonary nodule detection. Brit. J. Radiol. 79, 342–346. 10.1259/bjr/13453920 16585729

[B44] Metz C. E. (1978). Basic principles of ROC analysis. Semin. Nucl. Med. 8, 283–298. 10.1016/S0001-2998(78)80014-2 112681

[B45] Nakashima R. Kobayashi K. Maeda E. Yoshikawa T. Yokosawa K. (2013). Visual search of experts in medical image reading: the effect of training, target prevalence, and expert knowledge. Front. Psychol. 4:166. 10.3389/fpsyg.2013.00166 23576997 PMC3617447

[B46] Nakashima R. Watanabe C. Maeda E. Yoshikawa T. Matsuda I. Miki S.. (2015). The effect of expert knowledge on medical search: medical experts have specialized abilities for detecting serious lesions. Psychol. Res. 79, 729–738. 10.1007/s00426-014-0616-y 25269540

[B47] Nakashima R. Yokosawa K. (2013). Visual search in divided areas: dividers initially interfere with and later facilitate visual search. Atten. Percept. Psychophys. 75, 299–307. 10.3758/s13414-012-0402-0 23197334

[B48] Oestmann J. W. Greene R. Kushner D. C. Bourgouin P. M. Linetsky L. Llewellyn H. J. (1988). Lung lesions: correlation between viewing time and detection. Radiology 166, 451–453. 10.1148/radiology.166.2.3336720 3336720

[B49] Pelli D. G. (1997). The videotoolbox software for visual psychophysics: transforming numbers into movies. Spat. Vis. 10, 437–442. 10.1163/156856897X00366 9176953

[B50] Potter M. C. Faulconer B. A. (1975). Time to understand pictures and words. Nature 253, 437–438. 10.1038/253437a0 1110787

[B51] Rousselet G. A. Fabre-Thorpe M. Thorpe S. J. (2002). Parallel processing in high-level categorization of natural images. Nat. Neurosci. 5, 629–630. 10.1038/nn866 12032544

[B52] Sagi D. Julesz B. (1985). Fast noninertial shifts of attention. Spat. Vis. 1, 141–149. 10.1163/156856885X00152 3940055

[B53] Schwaninger A. Hardmeier D. Hofer F. (2005). Aviation security screeners visual abilities & visual knowledge measurement. IEEE. Aero. El. Sys. Mag. 20, 29–35.

[B54] Shapiro K. L. Arnell K. M. Raymond J. E. (1997). The attentional blink. Trends Cogn. Sci. 1, 291–296. 10.1016/S1364-6613(97)01094-2 21223931

[B55] Shapiro K. L. Raymond J. E. (1989). Training of efficient oculomotor strategies enhances skill acquisition. Acta Psychol. 71, 217–242. 10.1016/0001-6918(89)90010-3 2816474

[B56] Treisman A. (1988). Features and objects: the fourteenth bartlett memorial lecture. Q. J. Exp. Psychol. 40, 201–237. 10.1080/02724988843000104 3406448

[B57] Treisman A. Gelade G. (1980). A feature integration theory of attention. Cogn. Psychol. 12, 97–136. 10.1016/0010-0285(80)90005-5 7351125

[B58] Tversky B. Sherman T. (1975). Picture memory improves with longer on time and off time. J. Exp. Psychol. 104, 114–118. 10.1037/0278-7393.1.2.114 1141825

[B59] Vickery T. J. King L. Jiang Y. (2005). Setting up the target template in visual search. J. Vis. 5, 81–92. 10.1167/5.1.8 15831069

[B60] Voss J. F. Vesonder G. T. Spilich G. J. (1980). Text generation and recall by high-knowledge and low-knowledge individuals. J. Verb. Learn. Verb. Behav. 19, 651–667. 10.1016/S0022-5371(80)90343-6

[B61] Warm J. S. Parasuraman R. Matthews G. (2008). Vigilance requires hard mental work and is stressful. Hum. Factors 50, 433–441. 10.1518/001872008X312152 18689050

[B62] Wolfe J. M. (1998). Visual search, in Attention, ed Pashler H. (London: University College London Press), 13–73.

[B63] Yantis S. Jonides J. (1984). Abrupt visual onsets and selective attention: evidence from visual search. J. Exp. Psychol. Hum. 10, 601–621. 10.1037/0096-1523.10.5.601 6238122

